# Avoidance of plants unsuitable for the symbiotic fungus in leaf-cutting ants: Learning can take place entirely at the colony dump

**DOI:** 10.1371/journal.pone.0171388

**Published:** 2017-03-08

**Authors:** Andrés Arenas, Flavio Roces

**Affiliations:** Department of Behavioural Physiology and Sociobiology, Biozentrum, University of Würzburg, Würzburg, Germany; Universidade de São paulo, BRAZIL

## Abstract

Plants initially accepted by foraging leaf-cutting ants are later avoided if they prove unsuitable for their symbiotic fungus. Plant avoidance is mediated by the waste produced in the fungus garden soon after the incorporation of the unsuitable leaves, as foragers can learn plant odors and cues from the damaged fungus that are both present in the recently produced waste particles. We asked whether avoidance learning of plants unsuitable for the symbiotic fungus can take place entirely at the colony dump. In order to investigate whether cues available in the waste chamber induce plant avoidance in naïve subcolonies, we exchanged the waste produced by subcolonies fed either fungicide-treated privet leaves or untreated leaves and measured the acceptance of untreated privet leaves before and after the exchange of waste. Second, we evaluated whether foragers could perceive the avoidance cues directly at the dump by quantifying the visits of labeled foragers to the waste chamber. Finally, we asked whether foragers learn to specifically avoid untreated leaves of a plant after a confinement over 3 hours in the dump of subcolonies that were previously fed fungicide-treated leaves of that species. After the exchange of the waste chambers, workers from subcolonies that had access to waste from fungicide-treated privet leaves learned to avoid that plant. One-third of the labeled foragers visited the dump. Furthermore, naïve foragers learned to avoid a specific, previously unsuitable plant if exposed solely to cues of the dump during confinement. We suggest that cues at the dump enable foragers to predict the unsuitable effects of plants even if they had never been experienced in the fungus garden.

## Introduction

Social insect colonies are able to flexibly respond to changing environmental conditions. The observed collective patterns result from the decisions of individual colony members, which are to some extent directed by strong and meaningful stimuli releasing innate behaviors. Moreover, decisions can be based on learned information [1–5]. In the foraging context, animals may associate odors, colors and other features of the food sources with the rewards found shortly before or immediately thereafter. Learning also occurs within the social context of the colony [6–8]. Social learning is extremely important to generate an adaptive collective response as workers can gather information about different foraging options and respond according to their own experience and colony needs [9]. The question of how this information is learned and conveyed among thousands of nestmates is particularly interesting in leaf-cutting ants (genus *Atta* and *Acromyrmex*, Hymenoptera: Formicidae), in which plant selection is not simple determined by the workers’ innate or learned preferences, but also by the requirements of the fungus that lives in symbiosis with the ants [10–11].

Leaf-cutting ants are Neotropical herbivores that selectively cut plants and carry the harvested fragments back to their nests [12–14]. Once inside, the plant material is processed into a substrate to rear a symbiotic fungus maintained in underground nest chambers that represents the main food source for the colony members [15]. Plant selection by foragers is not only based on their own preferences [16, 13, 17, 18] but also influenced by the effect the collected substrates cause on the fungus. If a collected plant proves to be unsuitable for the fungus, ants respond by interrupting foraging on that specific plant [19, 20]. Plant avoidance does not occur immediately but several hours after the incorporation of the unsuitable leaves into the nest, a response known as “delayed rejection” [21, 19, 20, 22, 18, 23, 24]. Delayed rejection involves the formation of long-term olfactory memory [25, 26]. Inside the nest, gardeners and midden workers do also respond to unsuitable plants by discontinuing the processing of leaf fragments in the fungus garden and by disposing of unprocessed and partially decomposed plant material into the waste chamber [27].

Fungus cultivation produces a considerable amount of waste due to the decomposition of the substrate. According to the ant species, waste is disposed of outside the nest or inside specific underground chambers (waste dumps) along with exhausted fungus, dead ants, soil particles, and other debris [28–31]. The transport, manipulation and spatial isolation of waste in a dump are thought to be adaptive responses aimed at reducing the spread of pathogens within the nest [32–35]. However, waste might also represent a source of information as it contains cues that enable recognition of the plant materials from which it originates [36]. We recently showed that naïve foragers learned to avoid plants unsuitable for the fungus even if they were not directly exposed to them or to their effects on the fungus. This occurred when waste particles from a subcolony previously fed unsuitable leaves were introduced into the fungus chamber of a naïve subcolony, indicating that cues that enable plant identification and cues or signals that inform about the state of the fungus were both present in waste particles. Waste disposal might therefore be an important mechanism that helps propagate information exclusively generated in the fungus garden to other parts of the nest and even to the outside. Under this assumption, cues that are relevant for making foraging decisions may accumulate over time at the colony dump. So far, whether foragers learn to avoid plants that are unsuitable for the fungus using information solely available at the dump remains unknown.

In the present study, we investigated whether ants are able to learn about the suitability of a given plant solely from cues present in the waste chamber. We evaluated plant avoidance in foraging ants that have never experienced the negative effects of an unsuitable plant in the fungus garden, but had access to the waste chambers of subcolonies that have been fed leaves of the unsuitable plant. We hypothesized that ants are able to learn about plant suitability even if relevant information is no longer available in the fungus garden. Then the following questions were raised: Can workers of *Acromyrmex ambiguus* learn to avoid plants unsuitable for the symbiotic fungus based on information only available inside the waste chamber? For how long can putative avoidance memories be retained by foragers? Do foragers form their memories inside the waste chamber on their own, or via interactions with midden workers? If so, how often and under which conditions do foragers visit the waste chambers?

To answer these questions, we performed three experiments. To evaluate whether foragers learn to avoid plants unsuitable for the fungus when information is solely available in the waste chamber, we exchanged the waste chambers, but not the ants, between naïve subcolonies and subcolonies that were fed fungicide-treated leaves. Plant preferences by foraging ants belonging to both groups were compared before and after the exchange in dual-choice tests. Preferences were compared again with those measured one week after the emptying of the waste chambers, in order to test whether the learned avoidance of a specific plant persisted in the long-term without any informational cue available at the dump. In the second experiment, we quantified the visits of foragers to the waste chamber by labeling ants that foraged outside the nest in previous days and by video recording the nest dump. We recorded their visit times to the dump and scored whether they performed waste-related tasks. Because both the probability to visit the waste chamber and the performance of waste-related tasks might change after the incorporation of unsuitable leaves, we comparatively analyzed these variables under three different experimental conditions: i) *no leaves offered in the foraging box* (subcolonies were deprived of leaves), ii) *leaves in the foraging box* (subcolonies were fed untreated leaves they experienced as suitable), or iii) *after being fed treated leaves* (subcolonies were also fed untreated leaves they previously experienced as unsuitable). In the third experiment, we addressed whether foragers were capable of learning to avoid a specific plant after being directly exposed to a waste chamber that contained waste from fungicide-treated leaves of that plant. To this end, we confined naïve foragers in the waste chamber of subcolonies that were fed fungicide-treated leaves and measured their foraging preferences in dual-choice tests before and after the confinement. Their responses were also compared to those of non-confined nestmates.

## Material and methods

### Preparation of subcolonies

Experiments were conducted with queenless subcolonies built from six large queenright laboratory colonies of *Acromyrmex ambiguus* collected in La Coronilla, Uruguay. This species is not protected under the Convention on International Trade in Endangered Species of Wild Fauna and Flora (CITES). Export permits were issued by the Departamento de Fauna de la Dirección General de Recursos Naturales Renovables, Ministerio de Ganadería, Agricultura y Pesca, Uruguay. Colonies were kept in a climatic chamber at 25°C, 50% air humidity and a 12 h:12 h light:dark cycle. Each subcolony contained about 1000 workers and 1000 cm^3^ of fungus (i.e., fungus plus gardeners within the matrix). Their nests were organized in three compartments made of transparent boxes (19 x 8.5 x 8.5 cm): the fungus chamber containing a single fungus garden, the waste chamber, i.e., the box where ants disposed of the waste, and the foraging box. The three boxes were connected to each other by clear PVC tubes (15 cm long, 1.27 cm outside diameter) and a “T” junction. Subcolonies received fresh firethorn (*Pyracantha*) leaves, diluted honey and water every day.

### Leaf suitability and delayed avoidance

To change the suitability of the leaves for the fungus, we infiltrated plant material with a Cycloheximide (CHX; Sigma-Aldrich, Deisenhofen, Germany) solution (0.03%, w/w) that impairs the symbiotic fungus [22]. Cycloheximide cannot be detected by ants, which can continue foraging CHX-treated leaves for several hours. We used both blackberry (*Rubus fructicosus*) and privet (*Ligustrum*) as carriers for the CHX solution. Leaves were first cut with a puncher into small disks (diameter 6 mm) and then infiltrated. Two hundred CHX-infiltrated leaf disks were then offered at once at the foraging box to induce delayed avoidance of the infiltrated plant species.

### Experiment 1: Exchange of waste chambers between naïve subcolonies and subcolonies fed fungicide-treated leaves

**Fig 1:**
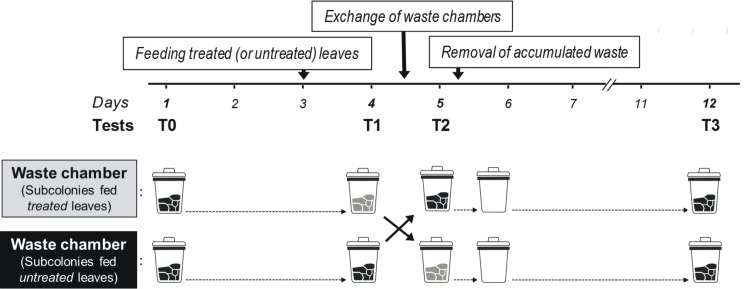
Schematic schedule of experiment 1 over the 12-days experimental period. Plant acceptance by single foraging workers was measured in dual-choice tests four times (from T0 until T3) to evaluate and compare the effects of three different treatments: *feeding treated (or untreated) leaves*, *the exchange of waste chambers*, and the *removal the accumulated waste* on plant avoidance. The content of the waste chambers at the time of each test is indicated for the group of subcolonies that have been fed either *treated* or *untreated* leaves. Waste colored with black originated from *untreated* leaves; waste colored with gray originated from *treated* leaves.

To investigate whether the avoidance of plants by foragers is mediated by the presence of the plant-related cues and cues or signals from the fungus in the nest dump, we exchanged the waste chambers of seven subcolonies that had been fed fungicide-treated privet leaves with the waste chambers of seven naïve subcolonies that had been fed untreated leaves. At the very beginning (at T0, day 1; Fig 1), before the exchange, initial plant preferences were measured in foraging ants from both groups of subcolonies. Preferences were quantified in dual-choice tests, where disks of privet leaves were simultaneously offered with disks of rose. Tests were carried out on a 3 x 3 cm platform located on the main trail, 1 m apart from the entrance of the nest, which offered four leaf disks (i.e., two leaf disks of each plant). Each test extended for 2 h, in which choices made by an average of 30.28 ± 0.22 foragers per subcolony were quantified for the 14 subcolonies. To guarantee a well-established foraging column, workers foraged on the arena for at least 1 h before the choice tests. Thereafter, single workers were allowed to reach the platform on which they could come into direct contact with the leaf disks of the two alternatives offered and make the choice. Once a single worker picked a disk up the disk was replaced and the loaded worker removed from the subcolony on its way back to the nest, and returned to the colony after the end of the tests. The intake of privet disks over the total intake of disks was used to quantify the standardized acceptance of privet, which ranged from 0.0 to 1.0 with a value of 0.5 indicating equal acceptance of the two offered plant species.

In order to obtain waste material containing cues that could mediate plant avoidance, one group of subcolonies was fed fungicide-treated privet leaves on day 3, and the other group untreated leaves (day 3; Fig 1). To confirm that subcolonies of the first group learned to avoid privet leaves treated with the fungicide, thus producing waste that is expected to contain avoidance cues from the plant and the damaged fungus, foragers´ preferences were measured the next day, for the two groups of subcolonies (at T1, day 4; Fig 1). Decisions made by an average of 28.71 ± 0.94 foragers per subcolony were recorded.

After determining the avoidance response towards privet in the subcolonies previously fed fungicide-treated leaves, hence the putative presence of plant avoidance cues in the dumps, the exchange of the waste chambers between treated and control subcolonies was made. All ants in the waste chambers were removed before the exchange. To remove as many ants as possible, the chambers were first disconnected from the nests and left opened for 20 min. Ants that remained in the chamber after this period were gently removed with forceps trying not to disrupt the structure of the waste pile. Because colony odours mediate nestmate recognition in ants [37], and waste might be recognized as foreign material by incoming ants, transfer of waste was always carried out between subcolonies built from the same large queenright laboratory colony (henceforth: sister subcolonies). By exchanging the waste chambers, we experimentally obtained naïve subcolonies with access to chambers containing waste from fungicide-treated leaves, and subcolonies that had experienced the effect of the fungicide directly in the fungus garden, yet with dumps that contained waste originated from untreated leaves. The effect of the exchange of waste chambers on individual foraging preferences was tested 16 h later (at T2, on day 5; Fig 1). This time was chosen because recent evidence indicated that waste disposal increased between 16 h and up to 28 h after a colony is fed fungicide-treated leaves [36]. In addition, changes in the fungus garden that induced plant avoidance can only be detected by workers for a relatively short time (1–2 days; [22], suggesting that avoidance cues learned by the ants are no longer noticeable in the fungus garden after this period. Although we could not rule out that waste particles originating from treated leaves could still have been disposed of after the exchange of the waste chambers, thus appearing in the recently-exchanged waste chamber of treated subcolonies at T2, previous evidence suggests that the disposal of waste particles in the dump is considerably reduced after 36 h [36]. At T2, decisions made by an average of 26.85 ± 1.63 foragers per subcolony were recorded.

Immediately after the tests at T2 were completed, the waste chambers of all subcolonies were emptied (Day 5, Fig 1). With no waste inside the chambers (hence no putative informational cues available), we evaluated if the avoidance memory established by the exposure to the waste chambers of subcolonies fed treated leaves was retained in the long-term. Thus, on day 12, i.e., 7 days later, we carried out the final, fourth test (T3). At T3, decisions made by an average of 25.75 ± 2.04 foragers were recorded only for 12 subcolonies, because one subcolony fed untreated leaves and one subcolony fed fungicide-treated leaves could not be tested successfully.

Since the homogeneity of variance assumption was met, repeated measures ANOVA was used to determine differences in acceptances among tests within the group of subcolonies fed fungicide-treated leaves, and within subcolonies fed untreated leaves. The treatment (feeding fungicide-treated versus untreated leaves) was the independent factor, and the testing event was the repeated measure. When significant interactions between these factors were detected, we applied simple effects to evaluate the influence of one factor separately for each level of the other [38]. Tukey tests were performed for *post hoc* comparisons for testing events within subcolonies fed fungicide-treated and untreated leaves.

### Experiment 2: Visits of foragers to the waste chamber

It is unknown whether foraging workers of *A*. *ambiguus* frequent the waste chambers, thus being able to acquire plant avoidance cues directly at the colony dump. To address this issue, waste chambers of six subcolonies were video recorded over 3 consecutive hours to quantify the visits of foragers to the dumps. Forty-eight ants per subcolony were first captured and labeled while they were foraging on the main trail. Ants were labeled with paint markers (Edding 750). From our experiments and other experiments done in our lab, there is no evidence that paint´s odors, if any, alter the behavior of labeled ants.

We used a two-color-marking code (by seven different colors) to enable individual identification of ants. Labeled foragers were identified on the video recordings to calculate how many of them visited the waste chamber. In addition, the duration of their visits was measured. We predicted a task shift from foragers to waste managers due to the increase in waste disposal after the offering of fungicide-treated leaves [36] and the resulting increase in task demands. Thus, we also scored the labeled ants that started performing waste-related tasks during their stays to calculate the proportion of foragers that switched to waste managers.

The variables indicated above were quantified under different experimental conditions: i) *no leaves offered in foraging box* (subcolonies were deprived of leaves), ii) *leaves in the foraging box* (subcolonies were fed untreated leaves they experienced as suitable) or iii) *after being fed treated leaves* (subcolonies were also fed untreated leaves they experienced as unsuitable 24 h before). We reasoned that under the first and second conditions, labeled ants might perform few visits to the waste chamber; on the other hand, we predicted a larger proportion of workers that would shift from foragers to waste managers in order to meet the increasing demand of waste handling after subcolonies were fed treated leaves.

### Experiment 3: Avoidance response of ants confined in the waste chamber

To investigate whether ants were able to learn to avoid a specific plant after being directly exposed to cues from both the plant and the damaged fungus at the dump, foragers from seven naïve subcolonies were transferred to and kept confined in waste chambers of seven subcolonies that had been fed fungicide-treated blackberry leaves. For the confinement, waste chambers were detached from the colonies and all the ants present therein were removed. Before the foragers were confined, their initial preference for blackberry leaf disks was evaluated in a dual-choice test (T_c_0) following the procedure used in Experiment 1. Blackberry and rose leaves were presented as choice. Foragers tested at T_c_0 were captured immediately after they made a decision, marked with a color dot on the gaster, and initially kept in a plastic box until 100–120 individuals foraging ants were collected and marked. Thereafter, all marked foragers were confined for 3 hours in the detached waste chamber, transferred back to their original subcolonies, and released in the foraging box.

Preferences of those foragers that had been confined were evaluated again (24 h later at T_c_1), together with the preferences of their unmarked (non-confined) nestmates. The test extended until at least 30% of the confined foragers were evaluated for the second time. It took about 2 h to attain such a percentage. Foraging preference was expressed as the standardized acceptance of blackberry leaves, calculated as the intake of blackberry disks over the total intake of disks (blackberry + rose) during the tests. Standardized acceptances of foragers before and after confinement, and of non-confined nestmates were compared using Wilcoxon Matched Pairs Test.

## Results

### Experiment 1: Exchange of waste chambers between naïve subcolonies and subcolonies fed fungicide-treated leaves

**Fig 2:**
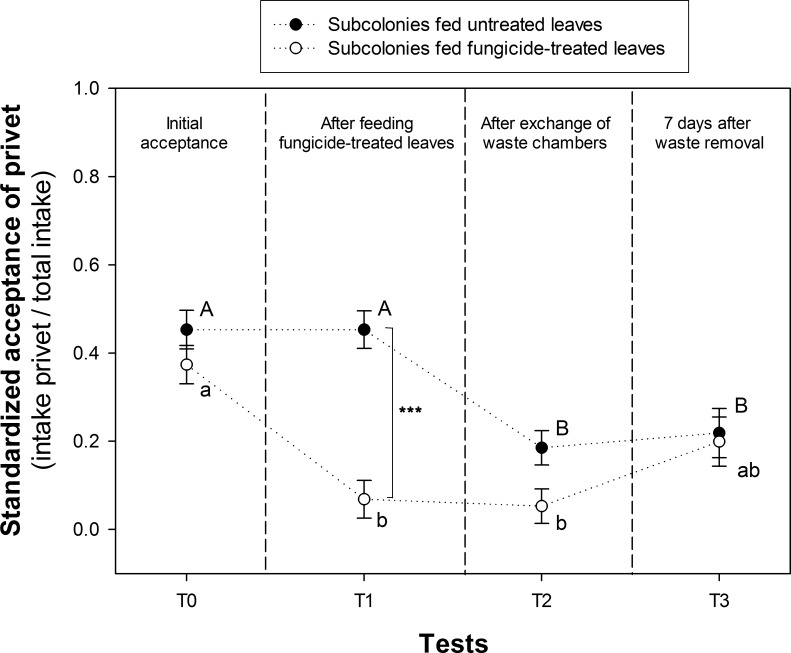
Plant acceptance by foragers after the exchange of waste chambers. Standardized acceptances of privet (intake of privet leaves / total intake) were recorded in individual dual-choice tests (privet vs. rose disks). Initial acceptance was quantified at T0. Standardized acceptance was recorded again at T1 (4 days later) after subcolonies of one of the two groups were fed fungicide-treated leaves of privet (white symbols). Standardized acceptances of the subcolonies fed fungicide-treated leaves and those fed untreated leaves (black symbols) were measured again at T2 (1 day later), after the exchange of the waste chambers. Finally, standardized acceptances were recorded 7 days after the removal of accumulated waste, at T3, to test memory retention. Dots represent the mean value and solid lines the S.D. from seven subcolonies with the exception of those at T3, which were only six. The number of workers tested per subcolony was indicated in the text. Different upper and lower case letters indicate statistical differences across testing events in subcolonies fed untreated or fungicide-treated leaves, respectively. Asterisks at T1 indicate significant differences between subcolonies fed untreated and fungicide-treated leaves; *** p < 0.001.

At T0, before any manipulation was conducted, foraging preferences by workers from the two groups of subcolonies were similar. As expected after one group of subcolonies experienced the unsuitable privet leaves on the previous day, preferences from the two groups became different at T1 (repeated measures ANOVA _treatment*testing event interaction_: F_3,30_ = 7.33, P < 0.001; simple effect analyses: F_1,40_ = 33.04, P < 0.001). Subcolonies previously fed treated leaves showed a strong decrease in the standardized acceptance of privet leaves from initial levels of 0.43 down to 0.07 (Tukey Test T0 vs. T1: P < 0.05, n = 401 choices; Fig 2, white symbols), indicating that ants successfully learned to avoid the previously treated leaves. On the contrary, subcolonies fed untreated leaves showed levels of acceptance (0.42) similar to those exhibited at T0 (0.34) (Tukey test T0 vs. T1: P > 0.05, n = 422 choices; Fig 2, black symbols).

At T2, the exchange of the waste chambers led to a strong decrease (from 0.42 to 0.16) in the standardized acceptance recorded in subcolonies now connected to waste chambers from subcolonies that had been fed fungicide-treated leaves (Tukey Test T1 vs. T2: P < 0.05, n = 378 choices; Fig 2, black symbols). The significant reduction in acceptance indicated that foragers learned to avoid the plant fed to subcolonies of the other group solely from information available at the dump. We could not detect that exposure to the waste chamber of subcolonies fed untreated leaves altered the response of subcolonies fed fungicide-treated leaves, which had already experienced the effects of unsuitable leaves directly on their fungus garden (Tukey Test T1 vs. T2: P > 0.05, n = 422 choices; Fig 2, white symbols).

One week after the empting of the waste chambers (at T3), foraging preferences by workers from the two groups of subcolonies were similar. Subcolonies that learned to avoid leaves via the exchange of waste chambers continued to show a similar decreased acceptance as measured at T2 (Tukey Test T2 vs. T3: P < 0.05, n = 376 choices; Fig 2, black symbols), at levels significantly lower than those measured at the beginning of the experiment (0.22) (Tukey Test T0 vs. T3: P < 0.05, n = 367 choices; Fig 2, black symbols). At T3, acceptance of subcolonies initially fed treated leaves were slightly higher (0.19) but not significantly different from those measured at T2 (0.25), (Tukey Test T2 vs. T3: P > 0.05, n = 418 choices; Fig 2, white symbols). They were also not significantly different from those measured at the beginning of the experiment (Tukey Test T0 vs. T3: P > 0.05, n = 354 choices; Fig 2, white symbols).

Taken together, results showed that avoidance learning not only took place after feeding unsuitable leaves, but also after workers got access to dumps containing waste originating from fungicide-treated leaves. Access to the dump containing avoidance cues led to the formation of a strong long-term memory in naïve foragers, which influenced their preferences at least for one week. However, little can be said about the retention of the initial avoidance memories when experienced workers had access to a cue-free dump after the exchange of the waste chambers, since their preferences at T3 were not statistically different from those at T0, T1 and T2.

### Experiment 2: Visits of foragers to the waste chamber

More than one third (35.41%) of the ants identified as foragers on the previous days visited the waste chamber of their nests. The proportion of ants that visited the waste chambers and the duration of their stays did not differ according to the experimental condition (Table 1). Foragers spent on average 13 min 54 s inside the waste chambers of their nests, with some ants that remained uninterruptedly in the dump for 2.5 h or longer. In the condition with *“no leaves in the foraging box”*, no labeled ants were observed displaying waste-related tasks, and less than 2% of them displayed task-related behaviors in the condition “*leaves in the foraging box*”. On the contrary, a significantly higher proportion of labeled ants displaying waste-related tasks, averaging 11%, was observed in the condition “*after being fed treated leaves”* (Table 1, Least Significant Difference = 4.228).

**Table 1:** Visits of foragers to the waste chamber and their behaviors. Number of marked foragers visiting the waste chamber as proportion of their total number, recorded in independent assays under different experimental conditions. The duration of their visits and the proportion of foragers performing waste-related tasks are also indicated. All values are expressed as means +/- SE.

	Experimental conditions					
*Variables*	*No leaves in foraging box*	*Leaves in foraging box*	*After being fed treated leaves*	*Statistics (Friedman Test)*	*N*	*P*
*Ants visiting the waste chamber (%)*	33.68 ± 3.62	38.88 ± 2.62	33.67 ± 5.73	T^2^_2_ = 0.95	18	0.42
*Duration of stay (s)*	842.04 ± 115.45	891.97 ± 134.99	769.91 ± 185.75	T^2^_2_ = 0.17	15	0.849
*Ants handling waste (%)*	0 ± 0	1.74 ± 1.36	11.11 ± 3.30	T^2^_2_ = 11.67	18	*0*.*002*

Number of marked foragers visiting the waste chamber as proportion of their total number, recorded in independent assays under different experimental conditions. The duration of their visits and the proportion of foragers performing waste-related tasks are also indicated. All values are expressed as means +/- SE.

### Experiment 3: Avoidance response of ants confined in the waste chamber

**Fig 3:**
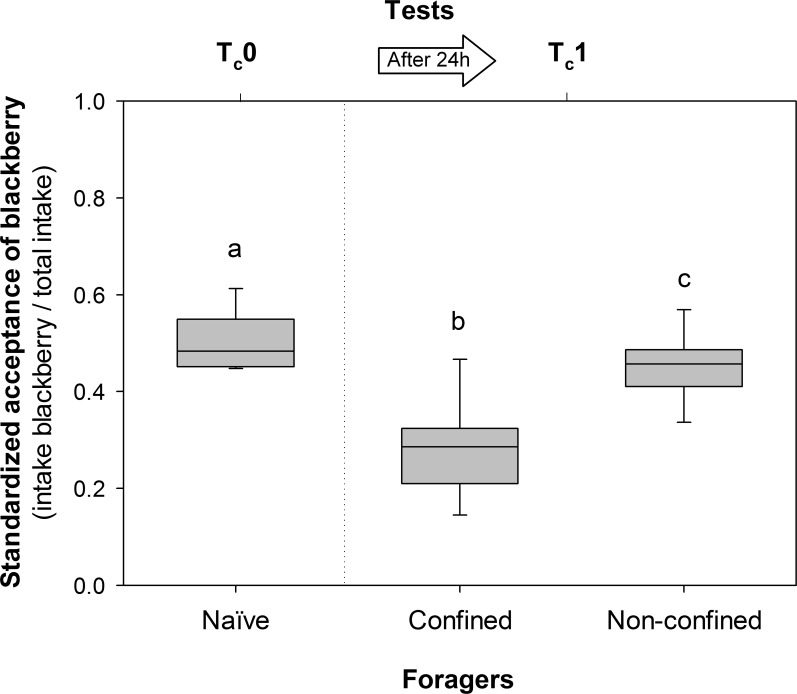
Plant acceptance of ants confined in the waste chamber. Standardized acceptance of blackberry (intake of blackberry leaves / total intake) was recorded in individual dual-choice tests (blackberry vs. rose disks). Initial acceptance of naïve foragers was recorded at T_c_0, before they were confined in the waste chambers of subcolonies that had been fed fungicide-treated blackberry leaves. Standardized acceptance of confined and non-confined nestmates were measured at T_c_1. The box plots show medians, quartiles and 5^th^ and 95^th^ percentiles from seven subcolonies. Different letters indicate significant differences among responses of naïve, confined and non-confined foragers after a Wilcoxon-Matched-Pairs Test.

Ants confined in the waste chambers of subcolonies that had been fed fungicide-treated blackberry leaves decreased their standardized acceptance for blackberry. From an initial value of 0.51 at T_c_0, acceptance of confined foragers decreased to 0.22 at T_c_1 (Wilcoxon Matched Pairs Test Tc0 vs. Tc1: Z = 2.366, p = 0.017, N = 7, n = 882 choices; Fig 3). Standardized acceptance of non-confined ants tested at T_c_1 was slightly but significantly lower than the acceptance they showed before as naïve foragers (Wilcoxon Matched Pairs Test Tc0 vs. Tc1: Z = 2.366, p = 0.017, N = 7, n = 1068 choices; Fig 3). Within the testing events performed at T_c_1, responses of confined and non-confined ants also differed (Wilcoxon Matched Pairs Test _confined vs non-confined ants_: Z = 2.197, p = 0.027, N = 7, n = 662 choices; Fig 3). The change in the foraging preferences before and after the confinement confirmed that foragers were able to learn avoidance cues directly from the waste chamber, without interactions with midden workers.

## Discussion

Here we showed that workers of *Acromyrmex ambiguus* learn to identify plants unsuitable for the symbiotic fungus entirely from information available in the colony dump. Even when plant choice under natural conditions could be much more difficult than in the special case of a dual-choice experiment in the laboratory, we demonstrated that acceptance of a specific plant decreased after waste chambers of naïve subcolonies and subcolonies fed fungicide-treated leaves of that plant were exchanged. A similar response was obtained when we confined foragers in the waste chamber of subcolonies fed fungicide-treated leaves, indicating that foraging ants were able to gain information and to learn about the suitability of host plants directly from the waste pile.

Leaf-cutting ants are expected to learn to avoid unsuitable plants inside the fungus chamber [19, 20, 22, 25], where the noxious effect of the harvested substrates for the fungus must be notorious. Due to the intimate contact gardeners have with the fungus, they are likely to associate plant-related cues with the deleterious effects of the incorporated plant on the fungus locally, as soon as changes in the state of the fungus took place. Gardeners themselves are active in plant avoidance as they discontinue the processing of noxious leaf fragments that may have qualified as suitable for foragers before their incorporation into the fungus garden [27]. It has been suggested [20] that experienced gardeners may in addition be in charge to inform nestmates with fewer opportunities to learn plant suitability inside the fungus chamber. Foragers, for example, are expected to spend a considerable amount of time outside the nest or in its periphery, yet it is unclear whether they also spend time inside the fungus chambers, for activities such as feeding or resting, whist they may have the opportunity to learn the avoidance cues. In a similar way, midden workers were longer considered as a worker group only active in the waste chambers and were not expected to visit the fungus gardens [33, 34]. However, recent experimental evidence indicates that both foragers and midden workers are well informed about the suitability of host plants and contribute to the “plant quality control” [27]. We recently observed that midden workers recovered fresh leaf fragments placed in the waste chamber to the garden, yet they did not recover fragments from plants they previously experienced as unsuitable [27]. Although we cannot rule out that workers performing tasks other than fungus tending could learn about plant suitability in the fungus garden, we suggest that avoidance learning does not necessarily require direct interactions with the fungus garden, and that it can entirely take place outside the fungus chamber too.

Refuse disposal is a common task among leaf-cutting ants [32, 39, 29] that avoids accumulation of waste in the garden and might help to propagate information about unsuitable host plants across the nest. It is reasonable to think that soon after the ants recognize that the fungus is growing on an unsuitable plant, they remove the garden as waste. We have recently demonstrated that waste particles newly removed from the garden contain cues that enable plant recognition and cues or signals that resemble the impairment of the fungus [36]. In particular, we observed that volatiles of the waste mediated the identification of unsuitable host plants. By scenting and transferring waste particles from subcolonies that had been fed either fungicide-treated or untreated leaves, we observed that the recall of olfactory memories formed through the exposure to scented waste was sufficient for the foragers to recognize and to avoid the unsuitable plant outside the nest[36]. Therefore, foragers might have the possibility to learn about the suitability of host plants in three different locations: inside the fungus gardens by directly assessing the state of the fungus, along the underground nest tunnels by interacting with waste-loaded nestmates, and in the waste chamber, as we showed in the present work.

Avoidance responses in leaf-cutting ants have been shown to be long-term. Foragers from field colonies of *Atta colombica* took up to 18 weeks to accept again the plants that proved noxious for the fungus [18]. If accumulation of waste in the nest dump contributes or not to the observed long-term response remains elusive. One would expect waste-related cues to diminish with time as the waste deteriorates and fresh waste from other plant sources accumulates in the dump. Thus it is tempting to speculate that cues that lead to plant avoidance remain in the waste chamber only for some time, depending on the waste turnover and degradation rates. With cues available for days or even weeks, waste deposits could act as a source of information for at least two worker groups. For experienced foragers, which could update their avoidance memories, and for naïve ants that could establish memories for unsuitable plants they have never experienced on the fungus garden by themselves. Up to now, our experiments showed that long-term memories formed at the dump last for at least 7 days without any update of information coming from the fungus garden or the dump. However, more experiments are needed to clarify the influence of cues present in the waste deposits on the establishment and maintenance of long-term foraging preferences.

In our laboratory subcolonies composed of approximately 2000 workers, about 35% of the labeled ants that foraged on the previous days were seen inside the waste chamber. While the small colony size may have influenced that proportion, the observation that foragers frequent the waste chambers is at odds with previous ideas about how leaf-cutting ants keep nest sanitation. Division of labor between ants working in the garden and in the waste dump was repeatedly reported as hygienic behavior [32, 33, 40, 41]. A study in *Atta colombica* further suggested that foragers do not frequent the waste deposits [34], and that waste management at the deposits was performed by old workers that remained there and were not allowed to enter the fungus chambers. Recent observations in midden workers of *A*. *echinatior* and *A*. *sexdens rubropilosa*, however, indicate that their behavior is more flexible than first thought. They do not remain restricted to the waste deposit. Instead, they perform many other activities, such as foraging or fungal care if conditions within the nest environment change [30, 42]. Although a separation between ants working inside and outside the waste deposit might be adaptive by reducing the spread of disease and pathogens present in the waste material [33, 34], we did not find evidence to support such separation in *A*. *ambiguus*. Indeed, more than 1/3 of the foragers labeled outside the nest were seen at the waste chamber despite the risks of colony contamination. In addition, midden workers of *A*. *ambiguus* were observed to move between the waste and fungus chambers and to recover fresh leaf fragments deposited in the dump depending on their previous experience [27].

One might argue that visits of foragers to the waste chamber were an experimental artifact resulting from the arrangement of the nest chambers, or because of the relatively small size of the laboratory subcolonies. However, it is worth mentioning that ants showed clear-cut orientation responses and discriminated between the ways leading to the different compartments of the nest, i.e., the fungus and the waste chambers, as also known for *Atta cephalotes* [43]. With certainty, we have never observed returning loaded foragers that failed to find the direct way from the foraging arena to the fungus chamber, and erroneously entered the waste chamber. Even though we cannot rule out that some foragers may have just reached the waste chambers accidentally, especially when no foraging took place, observations indicated that they were not reluctant to enter the chamber and to move on the dump. Unfortunately, little is known about the nest architecture of the species *A*. *ambiguus* [44] and how workers move through the underground nest tunnels to dispose of the waste particles. In general, colonies of most *Acromyrmex spp*. deposit the waste in underground chambers [45, 46, reviewed by 31], also *A*. *ambiguus* colonies [47]. The disposal of waste in underground chambers may delay its decomposition, as compared to waste exposed to open-air conditions, and thus enable learning by ants because of the conservation of waste volatiles for longer periods. It is therefore tempting to speculate that foragers from those *Acromymrex* species that have external dumps may rely less on the colony waste as a source of information about plant suitability than foragers from species having underground dumps. In comparison, leaf-cutting ants of the genus *Atta* build larger and much more complex nests than those of *Acromyrmex* leaf-cutting ants. Colonies of most *Atta* spp. deposit their waste in huge underground chambers [48, 49, 28, 50]. The extent to which *Atta* foragers rely on cues from the colony dump to form plant-avoidance memories remains elusive, as well as the possibility that sanitation strategies vary with colony size [51].

We estimated that about 11% of the foraging ants switched tasks and began managing waste after subcolonies were fed fungicide-treated leaves. Transition from one task to another is considered an important mechanism for the decentrally controlled organization of insect societies. At the colony level, the number of workers engaged in various tasks can be adjusted as needs and conditions change [52–54, 30]. We demonstrated that both the production and disposal of colony waste largely increased after the incorporation of unsuitable leaves [27, 36]. To meet the demands for handling high volumes of waste, unemployed ants or even ants engaged in other tasks are expected to get involved in waste-management duties. Consistent with this reasoning, our results showed that the switch from foraging to waste-related tasks occurred *after* the offering of fungicide-treated leaves but not *before*. In this scenario, task switching might be an adaptive response to bring more nestmates into contact with waste particles that contain avoidance cues, facilitating the propagation of relevant information about plant suitability. The probability of switching the task from foraging to waste-related duties may also depend on the size of the colonies. Workers in less populous colonies, like those of *Acromyrmex* species, may be more flexible in task allocation and more risk-prone than those of the large colonies of *Atta* species, thus visiting more often the waste deposits to gather information or to perform waste-related tasks. Further investigations should address whether the probability of task switching in workers changes according to colony size.

Learning to avoid plants that are unsuitable for the symbiotic fungus does not necessarily need a direct assessment of the effects of that plant on fungus growth. Foragers that had experienced the negative effects of an unsuitable plant neither in the garden nor in the dump might be influenced in their choices by interactions with experienced nestmates. In this regard, we showed that non-confined foragers slightly but significantly decreased their acceptance for the specific plant that only their nestmates initially experienced during the confinement in the waste chamber. This observation is consistent with previous results from [55], who observed changes in plant preferences when foragers were presented with a dual-choice test either alone, as a single forager, or in a group of several foragers. When one of the offered plant species was known as unsuitable and the other as suitable, more foragers “erroneously” accepted the unsuitable plant when foraging alone as compared to their responses as part of the group. These and our results suggest that preferences of naïve foragers are influenced by interactions with experienced nestmates. Similarly, the observed slight yet significant avoidance in non-confined foragers, which never directly experienced the cues from the waste chamber, may have resulted from interactions with their knowledgeable, previously confined nestmates during the shared foraging process. What kind of signals or cues mediates these interactions is completely unknown.

Up to now, it has been argued that avoidance memories that guide foraging can be acquired inside the fungus garden over a period of two days, in which cues from the impaired fungus, or plant identification cues, are still available [22]. Due to the enduring avoidance response by workers, which in some cases extended up to 30 weeks [21, 19], it was suggested that avoidance memories lasted for the complete lifetime of a forager [21, 19, 18]. Renewed acceptance of a previously-unsuitable plant at the colony level, therefore, was supposed to result from the turnover of ants in the colony. The observation that renewed acceptance of a previously-unsuitable plant occurred relatively suddenly in *Atta colombica* [18] was interpreted as an indication that experienced workers influenced the decisions of their naïve nestmates and precluded their plant acceptance over time, until the number of naïve workers overrode the number of the experienced ones. Considering that avoidance responses are also elicited by information entirely gained at the colony dump, as demonstrated in the present study, we wonder whether collective, long-term plant avoidance is brought about alone by lifetime memories in foragers, by the associations workers may form at the dump because of the long-lasting occurrence of plant avoidance cues, or by a combination of both.

## Supporting information

S1 Table Data used for calculations, tables and figures in the manuscript. Data for each table and figure are depicted in a separate sheet. (XLSX).
(XLSX)
